# Epstein Barr Virus Reactivation during COVID-19 Hospitalization Significantly Increased Mortality/Death in SARS-CoV-2(+)/EBV(+) than SARS-CoV-2(+)/EBV(−) Patients: A Comparative Meta-Analysis

**DOI:** 10.1155/2023/1068000

**Published:** 2023-01-31

**Authors:** Sivananthan Manoharan, Lee Ying Ying

**Affiliations:** ^1^Molecular Pathology Unit, Cancer Research Centre, Institute for Medical Research, National Institutes of Health, Ministry of Health Malaysia, Setia Alam, Shah Alam 40170, Selangor, Malaysia; ^2^Asia Metropolitan University, Bandar Baru Seri Alam, Johor Bahru 81750, Johor, Malaysia

## Abstract

Epstein–Barr virus (EBV) reactivation in acute-phase of COVID-19 disease was recently discovered but it is not clear in terms of degree of mortality caused, and this was the aim of the current study. Six databases and three non‐databases were thoroughly searched, independently. The articles related to non‐human study (abstract, *in vitro*, *in vivo*, *in silico*, case study, poster, and review articles) were excluded for main analysis. Four articles related to mortality linked to EBV reactivation were systematically identified and included in the qualitative and quantitative analyses. Based on proportional meta-analysis of 4 studies, 34.3% or 0.343 (95% CI: 0.189–0.516; *I*^2^ = 74.6) mortality related to EBV reactivation was identified. To address high heterogeneity, subgroup meta-analysis was carried out. Based on subgroup analysis, 26.6% or 0.266 (95% CI: 0.191–0.348; *I*^2^ = 0) with no heterogeneity was identified. Interestingly, in comparative meta-analysis, EBV(−)/SARS-CoV-2(+) patients had statistically lesser mortality (9.9%) than EBV(+)/SARS-CoV-2(+) patients (23.6%) where RR = 2.31 (95% CI: 1.34–3.99; *p* = 0.003; *I*^2^ = 6%). This finding is equivalent to the absolute mortality effect of 130 more per 1000 COVID-19 patients (95% CI: 34–296). Furthermore, based on statistical analysis, D-dimer was not statistically significantly different (*p* > 0.05) between the groups although studies have shown that D-dimer was statistically significantly different (*p* < 0.05) between these groups. Based on the inclusion and analysis of low risk of bias and high quality of articles graded with Newcastle–Ottawa Scale (NOS), when COVID-19 patients' health state is gradually worsening, EBV reactivation needs to be suspected because EBV reactivation is a possible marker for COVID-19 disease severity.

## 1. Introduction

Epstein–Barr virus (EBV) is a herpesvirus known to infect humans. After successfully invading the human body, EBV occupies B- and T-lymphocytes, epithelial, and muscle cells. Subsequently, the virus mainly becomes inactive. The reactivation of opportunistic viruses like EBV is associated with immunocompromised patients but also has been reported in patients with no earlier immunosuppression [1, 2]. A large impact of SARS-CoV-2 virus on immunological response in the COVID-19 patients has been reported [2]. It has been shown recently that the reactivation of EBV in recovered COVID-19 patients as one of the reasons behind long COVID symptoms [3]. Long COVID is coined when the former COVID-19 patients are still experiencing at least one symptom related to the disease after recovery from the acute phase infection [3]. On the other hand, the association of EBV reactivation in COVID-19 patients is still unclear and the investigations are ongoing. It has been reported that the ongoing COVID-19 patients reactivated with EBV and have higher percentage of respiratory failure than SARS-CoV-2 virus alone-infected patients [1]. This could be explained by the recent publication where the authors stated that EBV reactivation was linked with increased inflammation [4]. According to Gold et al. [3], the authors cited Chen et al. [4] as first to identify EBV reactivation during acute phase COVID-19 disease in Chinese patients. The study population of both Chen et al. [4] and Xie et al. [1] was from Wuhan, China where the COVID-19 pandemic first started. Several studies have reported mortality related to the reactivation of EBV in COVID-19 patients, but it is not fully clear due to the conflicting number of events between studies. To address this knowledge gap, we had conducted a systematic review and proportional/comparative meta-analysis to discover EBV reactivation related mortality in ongoing COVID-19 patients. Proportional meta-analysis is different from other types of meta-analysis because it involves single group data synthesis with no control. The results are presented as a percentage. This carries a unique challenge to the data synthesiser. Furthermore, there is no precise assessment for heterogeneity in proportional meta-analysis but *I*^2^ measure is taken into consideration. In proportional meta-analysis, *I*^2^ value is usually high. High *I*^2^ value does not necessarily mean the data are inconsistent. A conservative way of data interpretation is required to interpret the heterogeneity in proportional meta-analysis. Moreover, Egger's test, Begg's test (for publication bias), and funnel plots are not recommended for proportional meta-analysis. Instead, the results need to be interpreted qualitatively. It is possible to conduct such tests but no evidence stating the proportional data are sufficiently adjusted for the abovementioned tests [5]. To the best of our knowledge, this is the first meta-analysis related to mortality caused by EBV reactivation in COVID-19 patients.

## 2. Materials and Methods

In current meta-analysis, Preferred Reporting Items for Systematic reviews and Meta-Analyses (PRISMA) guidelines was followed to develop the manuscript. The current review's protocol was not prepared and registered. The authors followed Assessing the Methodological Quality of Systematic Reviews (AMSTAR) guideline while preparing the manuscript [6]. There were some challenges to follow this guideline since there are no proper controls and comparators available in 2 of the included articles/studies. This is because the main focus of the studies was to identify the amount/percentage of EBV reactivation in COVID-19 patients. Mostly, it was related to a single-arm study.

### 2.1. Research Questions

In current analysis, 3 research questions were addressed as follows:

- What is the percentage of mortality in EBV-reactivated COVID-19 patients?
- Does EBV reactivation significantly increase mortality in COVID-19 patients?
- Does D-dimer statistically significantly increase in hospitalized COVID-19-positive EBV-reactivated group when compared to the control group?

### 2.2. Search Strategies, Inclusion Criteria, Article Eligibility Criteria, Data Charting Process, Risk of Bias, Meta-Analysis, Certainty Assessments, and Paired *t*-Test

The articles were searched in ScienceDirect (11 articles), PubMed/MEDLINE (34 articles), Google Scholar (32 articles), Scopus (21 articles), Publons (5 articles), Cochrane (0 article), preprint-Research Square/MedRxiv (0 article), ClinicalTrials.gov (0 article), and reference lists (1 article) with suitable keywords (EBV reactivation COVID-19; EBV reactivation mortality COVID-19 patients; Viral coinfection and its related mortality in COVID-19 patients). The data charting process including screening of titles, abstract, and text was carried out by 2 authors, independently.

The risk of bias (RoB) assessment was carried out according to Newcastle–Ottawa scale (NOS). The score of 6 and above is regarded as a low RoB/high quality article. The MA was carried out using JBI SUMARI software where proportion (Freeman–Tukey statistical approach) and comparative MA using dichotomous data type, relative risk, confidence interval (CI), and Mantel–Haenszel statistical method were used [7]. Data were pooled, respectively. A fixed-effect model was used when the data are homogeneous while a random-effects model was used if the data are heterogeneous. Heterogeneity was defined as significant when *p* < 0.1 or *I*^2^ > 50% [8]. The certainty assessments were done using GRADEpro GDT software [9]. To the best of our search and knowledge, only retrospective/observational type articles were available and included for qualitative and quantitative analyses. No randomized control trials were available. The authors speculated that since the work was mainly related to single arm study in most of the articles, no randomization and controls were needed. Based on this explanation, the finalized articles were included for qualitative and quantitative analyses. The inclusion criteria are as follows:

1. EBV tested in acute phase of COVID-19 disease in the patients;
2. Any type of human-related studies except case study and reviews;
3. Not necessarily need to report clinical efficacy as study outcomes;
4. The results in the articles must be related to EBV reactivation-related mortality in COVID-19 patients.
5. In the case of 2 viruses being reactivated in the same patient, these data were included if one of the viruses is related to EBV and if there is no way to exclude another unrelated virus.
6. The literature published in English (language restriction) between the years 2020 and June 2022.

The independent *t*-test for D-dimer was carried out using MedCalc software [10]. Test for normal distribution using D'Agostino–Pearson test with CI of 95% was conducted assuming equal and unequal variances (done for both). Furthermore, logarithmic transformation was not made. The outcome was reported based on the generated *p*-value.

## 3. Results

### 3.1. Study/Article Inclusion

**Figure 1:**
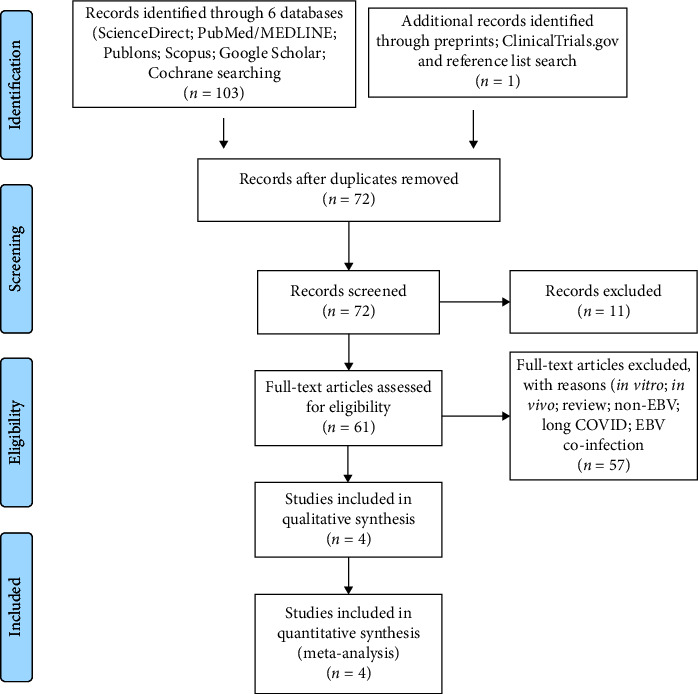
The PRISMA flowchart illustrating the systematic inclusion and exclusion process for the literature search related to EBV reactivation in COVID-19 patients.

Out of 104 shortlisted literature, 4 articles which met the inclusion criteria are included for qualitative and quantitative analyses as reported in Figure 1 PRISM flowchart [11]. Due to the very recent discovery of EBV reactivation, very limited availability of articles related to the mortality was found. On the other hand, several EBV reactivation-related articles without mortality were found. Moreover, many studies were done with small sample size numbers.

### 3.2. Characteristics of the Selected Articles and Risk of Bias (RoB) Assessment

In this review, 4 articles which met the inclusion criteria were included. The characteristics of the articles are listed in Table 1. Based on the NOS score for risk of bias assessment in Table 1, all included articles are from low-risk of bias category.

### 3.3. Meta-Analysis

**Figure 2:**
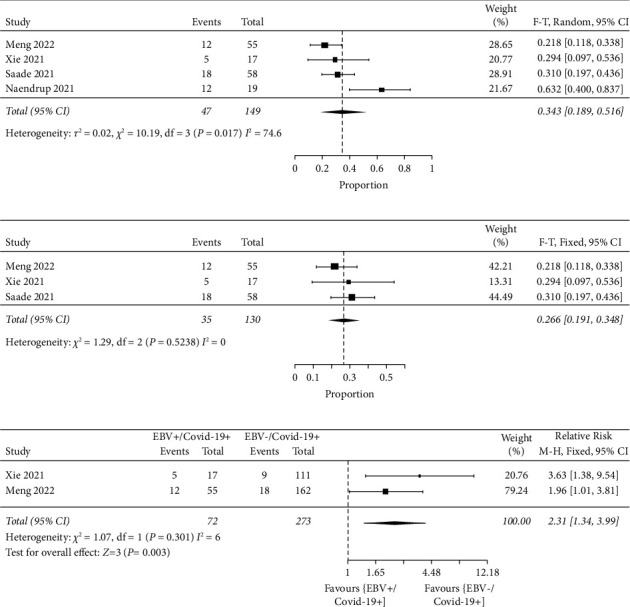
Forest plots of proportional and comparative MA, respectively. (a) Proportional MA using Freeman–Tukey (F–T) statistical approach to detect percentage of mortality in COVID-19 patients with reactivated EBV. The analysis was carried out using JBI SUMARI. The EBV reactivation data for Saade et al. [14] was extracted from the database provided by the authors. (b) Subgroup analysis to address high heterogeneity found in Figure 2(a). (c) Comparative meta-analysis using dichotomous data type, relative risk, confidence interval (CI), and Mantel–Haenszel (M–H) statistical method in COVID-19 patients with and without EBV.

To answer question 2.1 (i), proportional type of meta-analysis was carried out. This is because the available dataset (single group) in articles is not suitable for other types of meta-analysis, thus, proportional meta-analysis was chosen. Based on Figure 2(a), 34.3% or 0.343 (95% CI: 0.189–0.516; *I*^2^ = 74.6) of mortality was detected in EBV reactivated COVID-19 patients with significant heterogeneity. In proportional meta-analysis, high heterogeneity is expected and does not mean the studies are inconsistent. A conservative way of data interpretation is required to interpret the heterogeneity in proportional meta-analysis [5]. Based on Figure 2(a), out of 4 studies, Naendrup et al. [13] was poorly or did not overlap with total (95% CI). This represents significant inconsistency between different studies. Based on conservative heterogeneity data interpretation, the authors agreed that significant inconsistency in the included studies took place and this inconsistency was translated into high heterogeneity. The total and confidence interval of three out of 4 studies overlapped maximally. These 3 studies have a cumulative weight of 78.33%. Based on the traditional way of interpreting heterogeneity, the authors have almost 80% confidence that 34.3% mortality in EBV-reactivated COVID-19 patients reflects the actual scenario.

**Figure 3:**
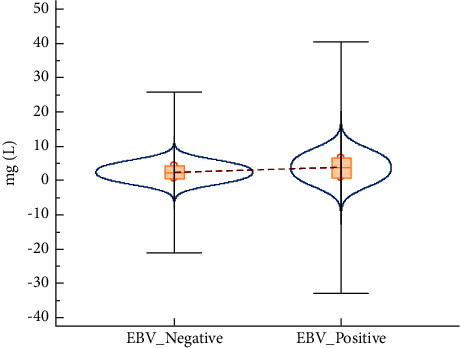
The violin plot of level of D-dimer in EBV(−)/SARS-CoV-2(+) and EBV(+)/SARS-CoV-2(+) group, respectively. The data were extracted from Xie et al. [1] and Meng et al. [12]. The D-dimer was not statistically significantly different between these 2 groups (*p* > 0.05).

Furthermore, the authors did subgroup analysis to address the high heterogeneity found in Figure 2(a). In Figure 2(b), based on subgroup analysis, 26.6% or 0.266 (95% CI: 0.191–0.348; *I*^2^ = 0) with no heterogeneity was identified. Based on subgroup analysis, with no heterogeneity found, it can be concluded that 26.6% of mortality is caused by EBV reactivation in COVID-19 patients. Interestingly, based on comparative meta-analysis of 2 studies in Figure 2(c), EBV-negative(−)/SARS-CoV-2-positive(+) group yielded statistically significantly lesser (in favour) mortality compared to EBV(+)/SARS-CoV-2(+) group where RR = 2.31 (95% CI: 1.34–3.99; *p*=0.003). The heterogeneity was insignificant with *I*^2^ = 6% and *p*=0.301. This shows that in the case of EBV(+)/SARS-CoV-2(+), more COVID-19 patients were dying than the patients with SARS-CoV-2 virus alone. In current analysis, 23.6% (EBV/SARS-CoV-2) and 9.9% (SARS-CoV-2 alone) of mortality/death were recorded. Based on Table 2, the overall certainty assessment was low. This is due to the nature of included studies where only observational studies were included. When the output in Table 2 was analyzed manually, both studies belong to the high-quality category. Basically, randomized controlled trials are preferred over observational studies [15]. Based on Table 2, it was revealed that the absolute mortality effect due to EBV reactivation in COVID-19 patients was 130 more per 1000 patients (95% CI: 34–296). According to Meng et al. [12] and Xie et al. [1], the mortality rate in EBV-reactivated COVID-19 patients was higher than non-EBV-reactivated COVID-19 patients although this finding was not statistically proven for Meng et al. [12]. EBV reactivation is probably a marker of severity of disease in SARS-CoV-2 virus-infected patients [12]. Interestingly, the D-dimer was statistically significantly increased in EBV(+)/SARS-CoV-2(+) patients than EBV(−)/SARS-CoV-2(+) patients in both studies [1, 12]. A surge of D-dimer in the patients infected by SARS-CoV-2 virus shows a hypercoagulable condition and consequently a high blood clotting possibility. The occurrence of acute respiratory distress syndrome (ARDS) is forecasted by the rise of D-dimer, which needs admission to the intensive care unit or might even cause death in serious patients [16]. Based on these interesting findings from both individual studies which came from Wuhan, China, an independent *t*-test was conducted to observe the statistical significance when both data were merged carefully. Based on Figure 3, D-dimer in ongoing COVID-19 patients with EBV reactivation was not statistically significantly different (*p* > 0.05) from ongoing COVID-19 patients with no EBV reactivation.

## 4. Discussion

The EBV can reactivate in people with impaired immune systems, as well as when physiological stressors such as an acute infection are present. In previous research, EBV reactivation was shown to occur during acute SARS-CoV-2 infection as indicated by the presence of detectable circulating EBV DNA or viral capsid antigen (VCA) IgM-positive [17]. In the current work, through meta-analysis, we have shown that EBV reactivation in COVID-19 patients increased the mortality. EBV viremia seems to relate to COVID-19 severity, an extended ICU stay, augmented interleukin-6 levels along with decreased CD8+ T and NK cell numbers [13]. Based on Table 1, we noticed that COVID-19 patients received glucocorticoid or dexamethasone as part of the treatment plan in all 4 studies. It has been shown earlier that 77% of seriously ill patients without immunosuppression have herpes virus reactivation. The corticosteroid use during the ICU stay has been identified as an independent risk factor for the disease in immunocompetent individuals [13]. Furthermore, the majority of EBV (58%) reactivations were identified in patients taking systemic corticosteroid treatment [13]. According to Naendrup et al. [13], additional 32% of patients had viral reactivation after taking systemic corticosteroids making 17/19 (90%) patients with EBV reactivation due to steroid treatment. Moreover, it has been shown that glucocorticoids, on top of inducing stress-related immune dysregulation, are able to mediate latent EBV reactivation via the induction of the BZLF1 gene [18]. According to Meng et al. [12], Herpesviridae reactivation was linked with old age, longer period of mechanical ventilation, an augmented intensive care unit length of stay, and also a lesser ratio of PaO_2_ to FiO_2_. Although the exact reason is unknown, the virus load is mostly reliant on patient immunity and immune suppression is largely related with EBV reactivation.

Interestingly, based on the analysis of the additional information supplied by Meng et al. [12] through e-mail, in general, we found out 18 out of 71 patients (25.35%) and 37 out of 146 patients (25.34%) had EBV reactivated in glucocorticoid- and nonglucocorticoid drugs-treated group, respectively. When analyzing dead and alive patients' cohorts for Meng et al. [12], we found out that out of 30 dead cases, 22 cases were given glucocorticoids with 8 EBV reactivated cases identified (36.36%). In the living patients' cohort, out of 187 patients, 49 received glucocorticoids with 10 EBV-reactivated cases identified (20.41%). Based on the percentages, the EBV-reactivated cases were higher in dead patients' cohort who received glucocorticoids compared to those in the living patients' cohort who received the similar drug. Besides, Saade et al. [14] reported that COVID-19 patients treated with dexamethasone in the ICU (44% versus 16%; *p* = 0.01) had viral reactivation although later after adjustment of confounding factors, Saade et al. [14] mentioned dexamethasone was no longer associated with viral reactivation. Since both studies are retrospective in nature, whether treatment with steroid drugs causes EBV reactivation needs to be addressed in a well-designed trial.

The current study has one major limitation known as the small number of sample sizes. Although the sample size in current MA is small (345 patients), the finding provides input and awareness regarding the statistically significant mortality rate in EBV-reactivated COVID-19 patients than that of non-EBV-reactivated COVID-19 patients. Furthermore, based on Table 2, by using this sample size, the absolute effect was determined and further reaffirmed that EBV reactivation increased mortality in COVID-19 patients. Moreover, the results need to be validated through randomized controlled trials since all the included studies in the current work are retrospective in nature. EBV reactivation needs to be suspected in the hospitalized patients with deteriorating health conditions because EBV reactivation is a possible marker for COVID-19 disease severity. Besides, as pointed out by Gold et al. [3], EBV reactivation too needs to be suspected in non-hospitalized long COVID sufferers.

## Figures and Tables

**Table 1:** Characteristics of the selected articles and risk of bias assessment.

Study	Study period	Country	Type of study	*n*	Age	+EBV, *n*	Sex (% male)	Treatments	MV, *n*	+EBV mortality, *n*	D-dimer(mg/L)	NOS
Xie et al. [1]	Jan–Mar 20	China	Retro	128	62	17	58.8	1–2 mg/kg intravenous glucocorticoids for 5–7 days in critically ill patients	NA	5	4.26 vs 6.67^*∗*^	**7**
Meng et al. [12]	Jan–Mar 20	China	Retro	217	54	55	37	(i) Ganciclovir (ii) Glucocorticoids (different study design to address mortality with or without ganciclovir treatment)	NA	12	0.56 vs 0.9^*∗*^	**7**
Naendrup et al. [13]	Mar 20-Mar 21	Germany	Retro	117	60	19	89	(i) Dexamethasone (6 mg/d) as part of the current COVID-19 treatment protocol (ii) Rituximab	NA	12	NA	**7**
Saade et al. [14]	Feb–May 20	France	Retro	100	60	58	75	(i) Dexamethasone (ii) Lopinavir/ritonavir (iii) Eculizumat (iv) Tocilizumab	37	18	NA	**7**

^*∗*^ = *p* < 0.05; MV = mechanical ventilation; NA=not applicable; n = number; d = day; NOS = Newcastle–Ottawa Scale.

**Table 2:** Certainty assessment of articles with GRADEpro GDT.

*Certainty assessment*							*No of patients*		*Effects*		Certainty	Importance
No. of studies	Study design	Risk of bias	Inconsistency	Indirectness	Imprecision	Other considerations	With EBV reactivation	Without EBV reactivation	Relative (95% CI)	Absolute (95% CI)		
*Mortality related to with and without EBV reactivation in COVID-19 patients*												
2	Observational studies	Not serious	Not serious	Not serious	Not serious	None	17/72 (23.6%)	27/273 (9.9%)	RR 2.31 (1.34 to 3.99)	130 more per 1,000 (from 34 more to 296 more)	⊕⊕○○ low	Critical

CI: confidence interval; RR: risk ratio.

## Data Availability

The data used to support the findings of this study are available from the corresponding author upon request.
